# Advancing treatment strategies for idiopathic normal pressure hydrocephalus: a systematic review on studies comparing ventricular and lumbo-peritoneal shunts

**DOI:** 10.1007/s10143-025-03582-2

**Published:** 2025-05-20

**Authors:** Matteo Palermo, Gianluca Trevisi, Francesco Signorelli, Francesco Doglietto, Alessio Albanese, Alessandro Olivi, Carmelo Lucio Sturiale

**Affiliations:** 1 Department of Neurosurgery, Fondazione Policlinico Universitario A. Gemelli IRCCS, Università Cattolica del Sacro Cuore, Rome, Italy; 2 Department of Neurosciences, Imaging and Clinical Sciences, G. D’Annunzio University, Chieti-Pescara, Italy; 3 Institute of Neurosurgery, Università Cattolica del Sacro Cuore, L.Go A. Gemelli 8, 00168 Rome, Italy

**Keywords:** Idiopathic normal pressure hydrocephalus, Ventriculoperitoneal shunt, Lumbo-peritoneal shunt, Ventricles

## Abstract

Idiopathic normal-pressure hydrocephalus (iNPH) is characterized by the clinical triad of gait disturbance, cognitive decline, and urinary incontinence. Cerebrospinal fluid (CSF) diversion is the gold standard treatment. Despite ventriculo-peritoneal shunt (VPS) is more commonly used, lumbo-peritoneal shunt (LPS) offers a minimally invasive alternative, raising questions about their relative efficacy and safety. A systematic review was conducted on multiple databases with a two-step selection process in order to exclude studies with insufficient data, irrelevance, and lacking of comparative analysis between the two procedures. From the included studies we comparatively analyzed preoperative clinical-radiological characteristics, surgical details and clinical-radiological outcome. We included 6 studies matching out inclusion criteria. Both VPS and LPS improved functional and cognitive performance. VPS provided faster symptoms relief, but has been related with higher risks of infection, whereas LPS showed a safer profile but required more frequent revisions due to mechanical issues. VPS and LPS are both effective treatments for iNPH. The choice of intervention should be tailored on the individual patient risk profiles, resource availability, and surgical expertise. Future research should focus on standardizing assessment scores, solve controversies, and evaluate long-term outcomes.

## Introduction

Idiopathic normal pressure hydrocephalus (iNPH) is a neurological condition characterized by a triad of symptoms: gait disturbances, cognitive impairment, and urinary incontinence [3, 27]. Radiological findings in iNPH are distinctive: ventriculomegaly (Evans Index > 0.3); acute callosal angle; disproportionally enlarged subarachnoid spaces (DESH). Although normal cerebrospinal fluid (CSF) pressure may appear normal on invasive measurement, iNPH is characterized by impaired CSF hydrodynamics [16, 18]. While the clinical presentation is well-defined, the underlying pathophysiology of iNPH remains poorly understood [7]. Moreover, not all patients presenting with the above clinical and radiological findings will respond to surgical treatment, and some ancillary tests are commonly used to select potential responders [4–7, 21, 32, 36]. iNPH is typically managed through CSF diversion by shunt intervention, despite intracranial pressure is usually within a normal range [10, 35]. Shunt procedures are designed to divert CSF from the cerebral ventricles to extracranial sites with absorptive capacity, most commonly the peritoneal cavity, thereby alleviating intracranial fluid accumulation.

The current standard options for iNPH include ventriculo-peritoneal shunts (VPS) and ventriculo-atrial shunts (VAS), draining CSF from ventricles to the peritoneum or right atrium, respectively. VPS is the most common type due to lower infection risk, although in some institutions VAS is considered as a first choice [15].

Lumbo-peritoneal shunts (LPS) is another possible technical choice diverting CSF from the lumbar subarachnoid space to the peritoneum. This type of shunt, for a long period almost completely replaced by the VPS, is recently experiencing a resurgence in popularity, particularly in Japan and other East Asian Countries especially for its procedural simplicity. In Japan, as of 2017, 55.1% of patients with iNPH underwent LPS procedures, surpassing the 43.2% who received VPS [25]. This trend contrasts with practices in Western countries, where VPS remains predominant. For instance, a study in the United States collecting data between 2007 and 2017, reported that only 15.5% of iNPH patients received LPS, while the majority underwent VPS [2].

This review aims to systematically evaluate the evidence comparing the clinical outcomes of VPS and LPS in patients with iNPH.

## Methods

**Fig. 1:**
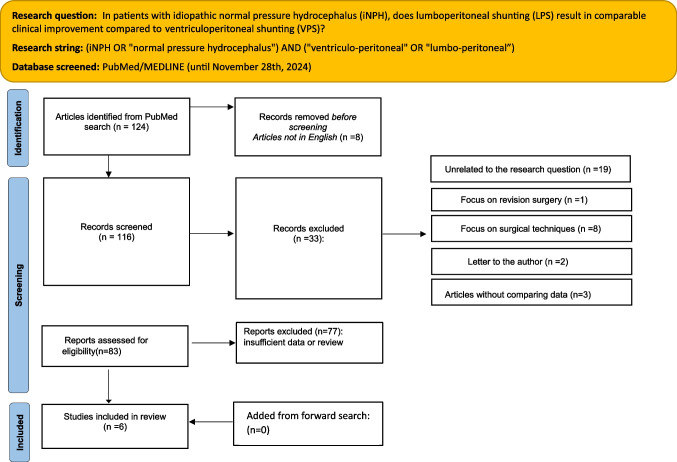
PRISMA 2020 flow diagram for new systematic reviews

This review was performed according to the PRISMA (Preferred Reporting Items for Systematic Reviews and Meta-Analyses) 2020 guidelines [28]. The PICO framework (*Population:* iNPH patients; *Intervention:* lumbo-peritoneal shunt (LPS); *Comparison:* ventriculoperitoneal shunt (VPS); *Outcome:* clinical improvement) was used to formulate the research question (Fig. 1).

### Search strategy

Two authors (GT and MP) performed a comprehensive search on PubMed/MEDLINE and Scopus databases to identify relevant studies comparing VPS and LPS using the search terms: (iNPH OR"normal pressure hydrocephalus") AND ("ventriculo-peritoneal"OR"lumbo-peritoneal"). The search was updated to November 28 th 2024, with no time limit. A forward search on references of the retrieved articles was also performed to increase the search power.

### Study selection

The search was limited to peer-reviewed studies published in English. Only papers that contained quantitative data were included. Other inclusion criteria were: papers comparing VPS and LPS; studies including more than 4 patients; follow-up of at least 6 months. Review papers and papers not presenting explicit data for each group (VPS and LPS) were excluded.

Two authors (CLS and MP) independently screened titles and abstracts of the articles retrieved by the search algorithm and selected studies according to the inclusion or exclusion criteria. After the exclusion of ineligible articles, full texts of the remaining studies were assessed for eligibility according to the same criteria (Fig. 1). Disagreements were resolved in a consensus meeting through a new reading of the article and collegial re-evaluation of the extracted data.

### Data extraction

For each eligible study, we extracted: author, year of publication, study design, total number of patients per treatment group, preoperative clinical and radiological characteristics, surgical details (including valve type, pressure setting, surgical complications, and re-operation rates), and clinical and radiological outcomes at a minimum 6-month follow-up. Clinical outcomes included global improvement and improvements in gait, cognition, and urinary function. Radiological outcomes were considered as ventricular size reduction.

### Presentation of data and statistical analysis

After a systematic review, we performed a meta-analysis when sufficient data were available from multiple studies for a specific outcome. This allowed for odds ratios (OR) calculation comparing LPS to VPS for a limited number of outcomes. Statistical analyses were performed using OpenMetaAnalyst software (http://www.cebm.brown.edu/openmeta/), based on R and funded by the Agency for Healthcare Research and Quality (Rockville, MD, USA).

### Quality assessment (risk of bias)

**Fig. 2:**
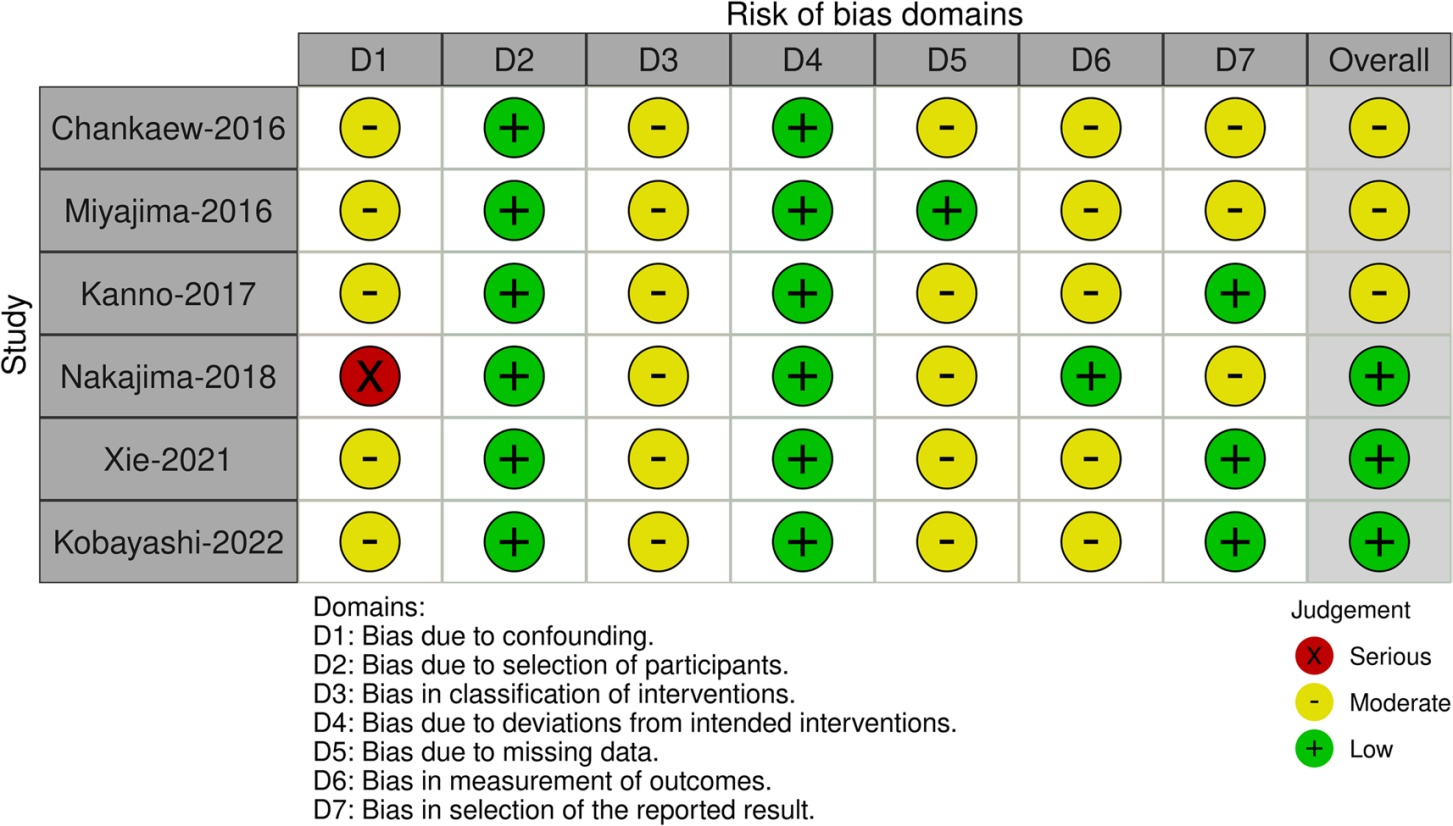
ROBINS-I V2 (Risk Of Bias In Non-randomized Studies – of Interventions, Vers. 2)

The ROBINS-I V2 (Risk Of Bias In Non-randomized Studies – of Interventions, Version 2) assessment tool along with the robvis application (https://mcguinlu.shinyapps.io/robvis/) were used to evaluate study quality through visual representations (Fig. 2).

## Results

The search algorithm initially retrieved 124 results. After applying predefined inclusion and exclusion criteria, 6 comparative studies evaluating VPS versus LPS were included in the final analysis. The selection process, detailing reasons for exclusion at each stage, is summarized in the PRISMA 2020 flowchart (Fig. 1). Risk of bias was assessed using the ROBINS-I V2 tool (Fig. 2), and study characteristics are summarized in Table 1.

**Table 1:** Summary of the included studies

Author and year	Study design	Patients Treated (n)			Primary Outcome (%)					Complications (%)				
		total	LPS	VPS	gait	urinary	cognitive	Global iNPH	Global mRS	Revision rate	Blockage	hemorrhage	migration	Over-drainage
Miyajima et al., 2016	P	187	87	100	NR	NR	NR	75% LPS vs 77% VPS	63% LPS vs 69% VPS	7% LPS vs 1% VPS	LPS > VPS	8% LPS vs 13% VPS (subdural)	6% LPS	24% LPS vs 8% VPS
Chankaew et al., 2016	P	53	20	33	NR	24.5%	NR	98.1%		NR	NR	1.9% VPS	NR	0/53 (0%)
Kanno et al., 2017	P	28	9	19	NR	NR	NR	71.5% no difference btw LPS and VPS	NR	NR	NR	NR	NR	NR
Nakajima et al., 2018	R	957	540	417	77.0% LPS vs 77.3% VPS	20.6% LPS vs 17.7% VPS	35.9% LPS vs 36.1% VPS	100% LPS vs 100% VPS	100% LPS vs 100% VPS	14.1% LPS vs 10.0% VPS	3.5% LPS vs 2.6% VPS	3.8% LPS vs 3.5% VPS (subdural)	NR	NR
Xie et al., 2021	R	76	38	38	9.5% LPS vs 10.5% VPS	NR	19.8% LPS vs 20.1 VPS	90% LPS vs 88.9% VPS	NR	2.5% LPS vs 2.8% VPS	2.5% LPS vs 5.5% VPS	0% LPS vs 11.1% VPS	NR	NR
Kobayashi et al., 2022	P	158	51	107	NR	NR	NR	NR	100% LPS vs 100% VPS	NR	2.6% VPS vs 3.5% LPS	NR	NR	NR

*LPS* lumbo-peritoneal shunt, *VPS* ventriculoperitoneal shunt, *RTC* randomized clinical trial, *P* prospective, *R* retrospective, *iNPH* idiopathic Normal Pressure Hydrocephalus scale, *NR* Not reported

### Qualitative analysis (systematic review)

The selected studies demonstrate that both LPS and VPS are effective surgical treatments for iNPH. However, due to substantial biases and deficiencies in the comparative data, definitive conclusions regarding significant differences in efficacy, complication rates, and overall safety profiles between these procedures are difficult to draw.

In 2016, as part of the SINPHONI-2 trial, Miyajima et al. compared LPS and VPS in iNPH patients [25] by using the iNPH grading scale, which measures the severity of symptoms focusing on gait disturbances, cognitive decline, and urinary incontinence. Both groups demonstrated substantial improvements in iNPH grading scale scores, and significant reductions in Evans’ index measuring the reduction in ventricular size. LPS patients showed a 75% improvement rate compared to 77% in the VPS group, with no significant difference in adverse events. An exception was seen in the VPS group, which carried a 1% risk of intraparenchymal hematoma, while no cases were observed in the LPS group.

In the same year, Chankaew et al. [9] reported a cohort of NPH patients undergoing both LPS and VPS, noting comparable improvements in the classical clinical triad across both groups. Moreover, this study highlighted a high prevalence of swallowing and speech problems in these patients, demonstrating significant improvements after shunt surgery in both groups. Importantly, this study found correlations between bulbar dysfunction and classical NPH symptoms, suggesting that the former should be also considered as a core symptom in diagnosis and management of NPH.

Additionally, Kanno et al. (2017) observed in DTI imaging improvements in fractional anisotropy (FA) values in the corona radiata, suggesting recovery of white matter integrity and reduction of the ventricular-dilation induced stress in shunt-responsive patients. On the other hand, non-responsive patients did not exhibit significant FA changes. They also observed that changes in FA and mean diffusivity (MD) were more evident in VPS patients, reflecting its higher association with a potential cerebral recovery. All in all, however, this study showed a global clinical improvement both in LPS and VPS patients measured as enhancing in iNPH grading and reduction ventricular size [17].

Nakajima et al. in 2018 also supported these findings, reporting an improvement in mRS scale of 63% for LPS and 69% for VPS patients at one year follow-up [26]. An additional analysis also showed similar improvements in gait, cognition, and urinary function between the two groups, although the frequency of shunt revisions was higher in LPS group (7% vs. 1%).

Similarly, in 2021, Xie et al. compared two cohorts of 38 patients treated with LPS and VPS, respectively, showing no significant difference in symptoms relief based on Kiefer’s Hydrocephalus Score (KHS), Mini-Mental State Examination (MMSE), and National Institutes of Health Stroke Scale (NIHSS). They observed an overall response rate (ORR) of 75% for LPS and 77% for VPS at six months follow-up. Furthermore, they noted significantly fewer complication rates in LPS group (20% versus 33% in VPS), especially due to the absence of ventricular puncture [37].

Additionally, all studies but for 1 [20] reported the use of programmable valves for both LPS and VPS group, of which only 1 reported specific values of pressure-settings [25] exclusively limited to the LPS group, with a mean of 13.5 ± 3.9 cm H_2_O.

Finally, Kobayashi et al. in 2022 confirmed comparable results between the two surgical techniques as regard to long-term improvement in gait, cognitive performance, and urinary function. Additionally, these authors explored risk factors for unfavorable outcomes, showing that hypertension and longer disease duration negatively impacted the outcomes in both groups [19].

Taken together, these findings demonstrate that LPS and VPS are associated with similar rates of clinical improvement. However, LPS apparently shows a lower complication rate, making it a viable option in patients at higher risk of procedural complications for VPS.

### Quantitative analysis (meta-analysis)

Due to data limitations, a meta-analysis was feasible only for outcomes reported in 4 out of 6 of the included studies. According to the global clinical outcome improvement, defined as a progression in at least one clinical domain (gait, cognition, urinary) at 6-month follow-up, LPS and VPS showed comparable outcomes (OR = 0.912; 95%CI:0.719–1.158; *p* = 0.4; I^2^ = 0%).

**Fig. 3:**
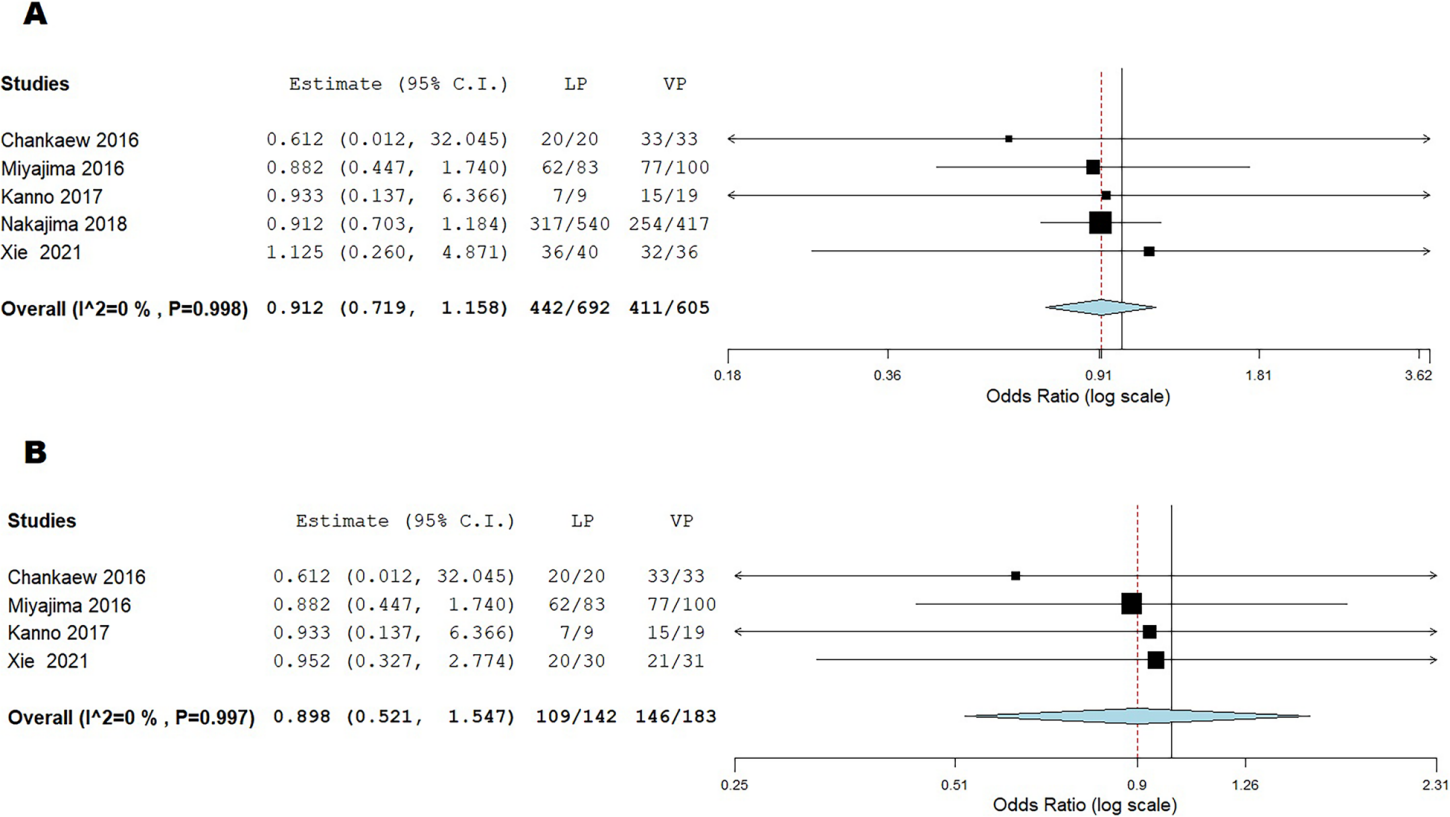
Quantitative analysis (meta-analysis). 3 A represents the global clinical outcome improvement, defined as a progression in at least one clinical domain (gait, cognition, urinary) at 6-month follow-up. 3B refers to gait control improvement in LPS and VPS groups

In particular, a gait control improvement was the most frequently reported good outcome, with no significant difference between LPS and VPS (OR = 0.898; 95%CI:0.521–1.547; *p* = 0.7; I^2^ = 0%—Fig. 3).

## Discussion

### Treatment efficacy across studies

This review provides evidence for a similar efficacy of both LPS and VPS shunts in improving iNPH symptoms. All six included studies demonstrated the efficacy of both procedures in relieving gait, urinary, and cognitive disturbances.

Among the 3 out of 6 studies focusing on clinical outcome, Xie et al. [37] noted a comparable cognitive recovery at MMSE and functional independence measure (FIM) scores in both groups; Nakajima et al. [26] observed a mean mRS improvement from 2.46 to 1.93 within one year regardless of shunt type; and, similarly, Miyajima et al. [25] reported mRS improvements in 63% of LPS and 69% of VPS patients.

Likewise, the prospective analysis by Kobayashi et al. compared a cohort of 51 newly diagnosed iNPH patients treated with LPS with 107 cases from a post-hoc analysis of the SIPHONI-2 trial treated with VPS revealed no apparent differences in mRS score, any iNPHGS sub-score or total score between the two groups. In addition, the analysis identified that greater preoperative symptom severity, particularly severe gait disturbance and poor functional status, was significantly associated with poorer postoperative outcomes, whereas disease duration was not. Notably, the tap test was effective in predicting postoperative gait improvement [19]. A 2024 meta-analysis of 4,811 iNPH patients found symptom improvement in 70–75% of cases with either VP or LP shunts. The study confirmed that LPS is non-inferior to VPS in terms of gait, cognitive, and continence improvements, with no significant difference in long-term outcomes over the past 15–20 years. This reassures that choosing LPS does not compromise symptomatic results, a conclusion supported by multiple studies [30].

However, not all included studies primarily focused on comparing clinical outcomes between VPS and LPS. For instance, one study examined the prevalence and impact of bulbar dysfunction in iNPH [9], another investigated white matter changes following shunt surgery [17] and a third explored the role of vascular risk factors in predicting surgical outcomes [19].

Nevertheless, these studies provided valuable clinical and radiological data on both LPS and VPS treatment groups, revealing comparable outcomes between the two shunt types. However, several studies have shown that a reduction in ventricular size after surgery does not always correlate with an improvement in clinical symptoms, and therefore, should not be considered a primary measure of outcome [23, 24].

Taken together, these findings suggest that shunt responsiveness is likely influenced more by patient selection than by the choice of procedure. In cases where response is incomplete, this may reflect the impact of comorbidities and the limitations of current diagnostic tools in accurately predicting outcomes [1, 4, 8, 13, 14, 22, 31, 32, 34, 36].

### Complications

Although overall complication rates were low, they varied significantly across studies. Miyajima et al. (2016) reported a higher rate of shunt revisions in the LPS group (7%) compared to the VPS group (1%), possibly reflecting intrinsic differences in device durability or function [25]. On the other hand, the unnecessary ventriculostomy in LPS patients determined a lower incidence of hemorrhagic complications.

In agreement, Xie et al. (2021) stressed the higher safety profile of LPS by comparing the risk intracranial hemorrhage and tube blockages between the two groups [37].

A recent meta-analysis found intracranial hemorrhages were twice as common with VPS (5% vs 2.4% with LPS). Seizures were also more frequent after VPS (~ 2.6% vs 0.2%) [15]. Moreover, any shunt over-draining CSF can cause chronic subdural fluid collections or hematomas; this occurred more often in VPS patients in some series, likely because siphoning effects are greater when the ventricular catheter is elevated above the abdomen. In contrast, an LPS inlet and outlet lie roughly at the same horizontal plane when upright, minimizing siphon-driven over-drainage [15].

However, it is important that newer programmable valves and anti-siphon devices have markedly reduced this problem. In fact, Miyajima et al. reported that with modern programmable valves, the overall serious and non-serious adverse event rates for LPS were statistically no different than for VPS at 1 year (22% vs 15% for serious events, *p* = 0.23).

The infection risk may even be lower with LPS, as a shorter hardware is placed. Pooled data show shunt infections in only 1.5% of LPS cases versus 5% with VPS (OR ≈0.3, *p* < 0.0001) [Ho]. Distal complications like peritoneal catheter infections or abdominal pseudocysts appear instead comparable between the two shunts [15]. Finally, LPS avoids direct brain injury, thus eliminating the risk of intracerebral hemorrhage, seizures, and central nervous system infections.

However, both shunt types share common general risks such as catheter migration, occlusion, infection and abdominal complications [26].

### Mechanical failures and revision rates

Malfunction, including occlusion, disconnection, and valve failure, is a critical concern in shunt surgery. In 2023, a systematic review by Ho et al. showed a significantly lower malfunction rate for LPS (3.99%) compared to VPS (8.31%) [15]. This likely reflects the absence of a ventricular catheter in LPS that can clog with choroid plexus or debris and the shorter length of the hardware. Ho et al. showed that across 25 included studies with 3654 patients, LPS was associated with roughly half the odds of mechanical failure compared to VPS.

However, some individual studies have reported higher revision rates with LPS. In the SINPHONI-2 trial, 7% of LPS patients required a shunt revision within 1 year versus only 1% of VPS [25]. The authors supposed a role of the learning curve of the procedure and the need to fine-tune valve pressure settings to avoid under- or over-drainage. Consistently, it has been observed that many LPS revisions occur early, being correlated to incorrect valve pressure or catheter positioning [33]. By comparison, long-term VPS revision rates is between 10–20% in most series [12, 25, 29]. Thus, current evidence suggests no gross difference in long-term shunt survival between the two techniques, although LPS might necessitate more frequent initial adjustments or revisions to optimize function. [11]. However, anti-siphon control and newer programmable valves with siphon guards have mitigated this issue [25].

### Factors influencing the choice of the procedure

The limited adoption of LPS in Western countries is probably due to factors such as historical preference, lower risk of displacement, concerns about CSF flow dynamics associated to LPS and surgical complications.

On the other hand, in Eastern countries, the preferable adoption of LPS is a practice dating back to the 1970 s for different reasons. Kazui et al. underlined that LPS should be the preferred option for iNPH given its minimal invasiveness, based on their controlled trial [25].

Therefore, nowadays the current difference stems from a greater level of experience and expertise in LPS placement in these regions.

However, the international literature supports that some patients’ factors represent the main guidance for choosing the preferred procedure. For example, severe lumbar spinal canal stenosis or prior lumbar surgery, represent strong criteria to prefer VPS over LPS. Conversely, for in patients with slit ventricles or with contraindication to cranial surgery, e.g. anticoagulation that cannot be reversed, a LPS is still preferable. Finally, another LPS advantage is the possibility to be performed in local anesthesia in elderly patients [11].

### Risk of bias and limitations

While we employed the ROBINS-I tool to systematically assess the risk of bias across included studies, it is important to discuss how these biases may have influenced our results. Most studies showed moderate risk in multiple domains, particularly in confounding (D1), classification of interventions (D3), and measurement of outcomes (D6). These issues could have led to inaccurate estimation of effect sizes, particularly if key confounders were not appropriately adjusted for or if intervention misclassification diluted the observed effects. The Nakajima-2018 study exhibited a serious risk of bias due to confounding, which may disproportionately impact our pooled estimates given the limited number of included studies. Furthermore, missing data (D5) and selective reporting (D7) were also judged to be of moderate concern in several studies, which may introduce attrition and reporting bias, respectively. These limitations reduce the internal validity of our findings and highlight the need for cautious interpretation, particularly in terms of causal inferences. The main difference among the reviewed studies lies in the variation of outcome measurement methods. Miyajima, Xie, and Nakajima assessed functional improvements using tools like the mRS and MMSE, while Kanno studied imaging signatures and biochemical markers to evaluate structural changes in the brain [17, 25, 26, 37]. To facilitate cross-study comparisons and meta-analyses, future studies should aim to standardize outcome measures.

Furthermore, most studies reviewed follow-up periods ranging from six months to one year, only used to evaluate short-term outcomes. Long-term studies are instead needed to assess the incidence of delayed complications, the durability of symptom relief, and the impact on the overall quality of life.

From the systematic analysis emerged the lack of a standardized protocol in selecting patients to their respective shunt group. Specifically, Miyajima et al. conducted a comparative study between patients in the SIPHONI-1 (prospectively enrolling patients who underwent VPS) and SIPHONI-2 (randomizing patients for LPS or conservative treatment) trials, Chankaew et al. and Kobayashi et al., selected the treatment strategy according to the shunt to the patient according to the patients’ wishes and conditions. The remaining studies did not explicitly mention the adopted protocol for their study.

Nearly all of the included studies did not evaluate the impact of different valve types or pressure settings on clinical outcomes between the two groups, with only one study serving as an exception [25]. While the study provides valuable insights into the use of programmable shunts for iNPH, there are several limitations to consider. First is the lack of comparison between different programmable valves, which could provide a clearer understanding of their varying effectiveness and suitability for individual patients. Secondly, the studies did not examine the influence of puncture site (frontal vs. parietal) on patient short-and long-term outcome. These factors could influence the success of shunt surgery, and their omission limits the ability to make definitive recommendations on the best technical approach for treating iNPH.

Therefore, despite the comparable effectiveness of the methods, the issue of patient selection remains a significant challenge. The variability in selection criteria introduces a bias that can compromise the objectivity of the results, underscoring the difficulty in achieving consistent and unbiased evaluations. Finally, it is important to note that these limitations reflect the current state of the literature rather than the quality of our review. We have critically assessed and synthesized the best available evidence, despite the inherent heterogeneity and data gaps across studies. This limits the generalizability of our findings and highlights the need for further high-quality studies to strengthen the evidence base. The small number of included studies also precludes robust subgroup analyses, which would have allowed a more nuanced understanding of effect modifiers and sources of heterogeneity. As a result, our conclusions should be interpreted with caution, recognizing that they may not fully capture the variability in clinical practice or patient populations.

## Conclusion

Insights from these studies reaffirm the efficacy of shunt surgery in managing iNPH, with both VPS and LPS offering comparable improvements in functional and cognitive outcome. The analysis of the comparative studies included in this review did not show the presence of a standardized protocol for shunt selection, but the decision is mainly based on patient’s preferences and the physician's judgment. Overall, although both techniques appear viable and may carry different profiles in terms of outcomes and complications, the current evidence remains inconclusive regarding their relative superiority. This highlights the need for a well-designed and adequately powered randomized controlled trial to directly compare the two approaches and provide clearer guidance for clinical decision-making.

## Data Availability

Raw data are available upon reasonable request.
